# Electrochemical tuning of the optical properties of nanoporous gold

**DOI:** 10.1038/srep44139

**Published:** 2017-03-09

**Authors:** D. Jalas, L.-H. Shao, R. Canchi, T. Okuma, S. Lang, A. Petrov, J. Weissmüller, M. Eich

**Affiliations:** 1 Hamburg University of Technology, Institute of Optical and Electronic Materials, Hamburg, Germany; 2 Hamburg University of Technology, Institute of Materials Physics and Technology, Hamburg, Germany; 3 Beihang University (BUAA), Institute of Solid Mechanics, Beijing, P.R. China; 4 ITMO University, St. Petersburg, Russia; 5 Institute of Materials Research, Helmholtz-Zentrum Geesthacht, Max-Planck-Strasse 1, Geesthacht, D-21502, Germany

## Abstract

Using optical *in-situ* measurements in an electrochemical environment, we study the electrochemical tuning of the transmission spectrum of films from the nanoporous gold (NPG) based optical metamaterial, including the effect of the ligament size. The long wavelength part of the transmission spectrum around 800 nm can be reversibly tuned via the applied electrode potential. The NPG behaves as diluted metal with its transition from dielectric to metallic response shifted to longer wavelengths. We find that the applied potential alters the charge carrier density to a comparable extent as in experiments on gold nanoparticles. However, compared to nanoparticles, a NPG optical metamaterial, due to its connected structure, shows a much stronger and more broadband change in optical transmission for the same change in charge carrier density. We were able to tune the transmission through an only 200 nm thin sample by 30%. In combination with an electrolyte the tunable NPG based optical metamaterial, which employs a very large surface-to-volume ratio is expected to play an important role in sensor applications, for photoelectrochemical water splitting into hydrogen and oxygen and for solar water purification.

Charge carrier injection or extraction is viable route to significantly modify the optical response of very thin layers. For example, in ref. 1 it was shown that a 10 nm thin ITO-layer is sufficient to build a modulator with 37% modulation depth for a wavelength of 1620 nm. The strong response was achieved by applying a voltage to the thin layer. This changed the charge carrier concentration and, along with it, the optical properties. Similarly, ref. 2 showed that by applying a voltage to a single layer graphene ribbon the optical transmission through the monolayer could be tuned by 10% in the terahertz and near-infrared region. Both of these concepts are limited to near-infrared and longer wavelengths. For higher frequencies (shorter wavelengths), beyond the plasma frequency of ITO and graphene, both material systems do not show the cooperative electron movements anymore which are typical to metals. The materials thus behave rather like dielectrics employing a limited displacement polarization in external electric fields. If one wants to extend the concept of tuning optical properties by charge injection to visible wavelengths, a material with its plasma frequency in the visible or ultraviolet region would be needed.

In principle, metals such as gold or silver have a suitable plasma frequency. But their already high density of charge carriers in the bulk is difficult to alter significantly by applying an external voltage. However, it is possible to strongly alter the surface charge carrier density of gold films by immersing them in an electrolyte and applying a voltage^3^,^4^,^5^,^6^,^7^,^8^. The accessible surface area is limited in this case. Our approach is to convert this strong surface charge carrier response into a bulk effect by structuring the gold on the nanoscale into sponge-like shape. In such nanoporous gold (NPG), the surface-to-volume-ratio is dramatically increased to values of typically 10^8^ m^−1^. The electrolyte can penetrate into the NPG and the electronic response of practically the whole NPG volume will be affected by strong changes of the charge carrier density in the thin gold ligaments. This makes the NPG-structure highly sensitive to external fields and to changes in the electrolyte properties, thus an attractive sensor platform. Structuring on the nanoscale is not only important to ensure the large surface to volume ratio but also to warrant that the optical wave sees an effective optical metamaterial. The latter means that the material properties can be averaged over the structure if the feature sizes are much smaller than the wavelength. If these conditions would not be fulfilled the NPG would strongly scatter light. Due to its large surface-to volume-ratio the interfacial area between a nanoscaled connected metal skeleton and the electrolyte is extremely large and makes this novel material a highly attractive base for plasmonically enhanced photocatalytic reactions, such as for water splitting into hydrogen and oxygen and for solar water purification^9^.

NPG is fabricated by selective dissolution of an alloy (dealloying). A network of uniformly distributed ligaments and pores results with length scales that can be adjusted between several nanometers and a few micrometers, depending on dealloying conditions^10^,^11^. NPG has already been shown to exhibit interesting optical properties and applications, such as surface-enhanced Raman scattering^12^,^13^, enhanced luminescence^14^,^15^, and has a distinctive transmission spectrum in the visible^16^,^17^.

We have recently shown that the optical properties of NPG can be qualitatively described by modeling the NPG as a cubic grid of crossing gold wires^18^. Figure 1(a) shows a simulated transmission spectrum of such a gold wire network. The transmission was first simulated assuming bulk gold material parameters and then, taking into account the experimental results, assuming a charge carrier density reduced by eight percent from the bulk value. Details on the simulation can be found in the Supplementary Information. The resulting spectral features stem from the interplay between the wires that are parallel to the optical polarization, which show metallic behavior, and the wires orthogonal to the polarization, which show dielectric behavior. At 500 nm wavelength the electric field averaged over gold and pore dielectric is close to zero. This gives rise to a peak in the imaginary part of the effective permittivity with the consequence of an increased absorption at that wavelength. Depending on the refractive index of the surrounding medium and the losses in the metal, this effect causes a dip or a plateau in the transmission spectrum, such as for the simulated model. The transmission experiments indicate that the transition from dip to plateau in the spectrum depends on the dealloying potential, thus on the ligament size. The ligament size is expected to slightly influence the material parameters of the nanoscale gold such as the effective loss, thus changes the shape of the plasmonic resonance spectrum. For longer wavelengths the material shows metallic behavior and can be understood as a diluted gold optical metamaterial. This can be clearly seen by comparing the field distribution at 500 nm to that at 950 nm as shown in Fig. 1(d) and (e). At 500 nm the field is localized with roughly the same field strength in the parallel and the orthogonal wires. In contrast to that, at 950 nm, the field is mostly localized in the parallel wires which leads to metallic behavior because these parallel wires connect the structure along the direction of polarization. When we compare the simulation to the experimental curves, we find that the experiment features a lower transmission. The reason for this is that our simple model of straight wires neglects the additional absorption and scattering of the more complex topography of NPG.

Electrochemical tuning of NPG in an electrolyte can lead to many interesting applications. One example would be the tuning of metamaterials. Especially in the visible range tuning mechanisms are lacking^19^. The electrochemical tuning of a metamaterial based on sub wavelength structures from solid gold has already been shown^20^ and a replacement of the solid gold with NPG would even enhance the effect. Another application is the monitoring of electrochemical processes through the change in optical properties^21^ as for example done for glucose detection^22^,^23^,^24^. Gold and other metals have already been used for optical electrochemical experiments in various shapes and forms. Among these are continuous metal films^3^,^4^,^5^,^6^,^7^,^8^, metallic nanoparticles^25^,^26^,^27^,^28^, nanoholes in metal films^29^,^30^, as well as metamaterials^20^,^31^,^32^. This large interest originates from the fact that metals are able to strongly concentrate light and by that allow for very localized and sensitive measurements.

Compared to the mentioned solid gold based approaches, NPG bears a number of important advantages: its fabrication requires no nanoscale lithography, the optical read out can be accomplished by a simple transmission measurement, and its porous structure allows for a high surface to volume ratio, thus for strong capillary forces. Further, the connected gold structure allows for simultaneous measurement of the electrical and optical properties. If one compares the transmission spectrum of the NPG model to that of an array of isolated gold nanospheres, one can see that the optical metamaterial consisting of plasmonic nanoparticles shows a strong narrowband dip in transmission close to its resonance whose spectral position expectedly depends on the carrier density. The main point is that, towards longer wavelengths, the structure becomes highly transparent and thus shows little to no dependence on changes of the carrier density. This is an immediate consequence of the fact that the isolated gold particles can provide their individual localized plasmon resonance contributions around 550 nm to the overall transmission spectrum, while they cannot contribute cooperative electron movement over larger scales. In contrast to that the gold network shows a broadband response in the red to infrared spectral region which stems from the connectedness of those wires parallel to the light polarization. This latter fact introduces cooperative electron movements just as in regular metals – just with a lower effective carrier density. This behavior can be seen in Fig. 1(e). At the longer wavelength of 950 nm the unconnected part of the structure extrudes the electric field and the light does not interact anymore with it which is comparable with the isolated gold particles. In the gold grid only the connected wires parallel to the light polarization interact with the light.

## Methods

As a basis for the NPG samples served ca. 200 nm thick, 6 Carat, i.e Au_25_Ag_75_ (mass%) white gold leafs (Wasner Blattgold) that were glued to 1 mm thick optical glass slides, using epoxy adhesive (Araldite LY564 with hardener Aradur 2954 (Huntsman), mixing ratio 3:1 by mass). The glue was cured at 80 °C for about 1.5 hours and then cured further at room temperature overnight. As the last step, samples were electrochemically dealloyed in 1 M HClO_4_ solution, using Ag/AgCl pseudo-reference electrode in the same solution and silver wire as counter electrode. Dealloying potentials were 700, 800 and 900 mV, samples of this work are denoted accordingly as 700, 800 and 900. Further details on the dealloying procedure may be found in ref. 33. Figure 2(a) shows a typical scanning electron micrograph of a NPG sample. The three different dealloying potentials of samples 700, 800 and 900 resulted in the mean ligament diameters, *L*, of 20(±5) nm, 15(±5) nm and 10(±5) nm, respectively. The corresponding surface-to-volume ratios (per volume of the solid), α, can be estimated as α = 4/*L*.

The setup for the *in-situ* transmission measurement is shown in Fig. 2(b). A quartz glass cuvette (Hellma Analytics) was filled with 0.7 M NaF electrolyte. The NPG sample was cleaned in ultra-pure water (Arium 611, Sartorius), immersed in the cuvette and connected to a potentiostat. All the potentials given here are relative to a Ag/AgCl pseudo-reference electrode in the same solution. For optical measurements the electrode potential, *E*, was stepped and normal incidence transmission spectra from 300 nm to 1200 nm were recorded at constant *E* after the electrode current became stationary. A UV-Vis-NIR spectrometer (Perkin Elmer Lambda-1050) was used. The transmission spectra were normalized with respect to the transmission through the glass substrate coated with an epoxy layer of identical thickness in the same solution and electrochemical cell. Further, the spectra below and above 800 nm were recorded with two different detectors. Because the samples were weakly scattering and both detectors had different sizes, the two detectors collected from slightly different solid angles thus registered slightly different fractions of the total transmitted signal. For this reason, the transmission measured by both detectors at 800 nm differed by a few percent. We corrected for this offset by scaling the longer wavelength part of the spectrum to match the shorter wavelength part of the spectrum at 800 nm, thus enforcing the whole spectrum to be continuous. Note that this implies an ambiguity of the absolute transmission level of a few percent. However, the comparison of a single sample at different tuning voltages is still unambiguous.

Our *in situ* experiments used aqueous solutions of NaF as the electrolyte, since the F^−^ anion forms a strongly bound hydration shell that minimizes chemical interaction off the anion with the gold surface. The Na^+^ cation does not interact since it forms an even more strongly bound hydration shell. During the first few cycles of the potential applied on the NPG the optical transmission underwent a non-reversible change^20^. This change is assigned to an enhanced mobility of gold atoms associated with a transient increase in surface diffusivity during the lifting of the oxygen adsorbate layer at the positive end of the potential scale^34^,^35^. We cycled the samples until the optical response showed a reversible behavior and then investigated the optical tuning.

The regime of electrode potentials was chosen so as to maximize the charge transfer while avoiding the evolution of hydrogen or oxygen at the lower or upper vertices of the potential scans, respectively. This limits the potential interval to *E* = −0.9 V … +0.9 V. Figure 3 shows a cyclic voltammogram typical of the reversible electrochemical cycles. The most obvious feature is the peak resulting from electrosorption of oxygen species at potentials *E* > 0.4 V during the positive going scans and the associated reduction peak during negative-going scans at *E* < 0.4 V. Small anion electrosorption peaks are also visible (presumably related to the presence of the epoxy glue), as is a significant Faraday current at *E* < 0. The Faraday current results from molecular oxygen impurities that cannot be avoided since the *in situ* cell is open to air. Superimposed to these extrinsic features is the intrinsic capacitive behavior of clean gold surfaces, which gives rise to the nearly constant current during positive going scans between 0 and 0.4 V.

## Results and Discussion

Figure 4 shows the transmission spectra of NPG with the potential changing in a large window including oxidation and reduction, i.e., from −0.9 V to 0.9 V for all three samples. In contrast to the measurement in Fig. 3, which is a dynamic measurement, the measurement for each potential was done after the charging currents were saturated. It can be seen that the change in the optical properties is strongly dependent on the polarity of the potential. For negative potentials the wavelength position of the transmission dip and the transmission change only little as compared to positive potentials, where a strong change is observed. This observation is consistent with the electrochemical signature: predominantly capacitive behavior with lesser capacity at potentials <0.4 V versus pseudo-capacitive behavior, with its stronger charge transfer associated with oxygen electrosorption, at the more positive potentials.

Since the transfer of electric charge scales with the area of surface, and since the specific surface area scales inversely with the structure size, the impact of the electrochemical potential on the optical behavior should be the strongest for the NPG with the smallest ligaments. This is exactly what is experimentally found. Specifically, consider Fig. 4(c) in comparison to (b) and (a). Here, (a) features the largest ligaments and (c) the smallest; and indeed the modification of the spectra is largest in (c).

It is also observed that the longer wavelengths are more affected by the change in potential compared to the shorter wavelengths. This observation has an obvious explanation: For the short wavelengths below 500 nm the optical properties of gold are governed to a large part by interband transitions^36^ which are not affected by a change of free charge density. By contrast, for the longer wavelengths gold can be described as a free electron gas (Drude model) whose properties strongly depend on the average number of mobile charge carriers. Since the charge carrier density is a property of the gold that is independent of wavelength we can use any part the spectrum to quantify it. Therefore, the change in number density (conduction electrons per volume), *n*, of these mobile charge carriers can be estimated by the wavelength shift of the dip near 500 nm:


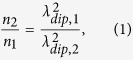


where *n*_1_ and *n*_2_ are two charge carrier densities and *λ*_*dip*,1_ and *λ*_*dip*,2_ denote the corresponding positions of the dip in transmission. A derivation of eq. (1) can be found in the Supplementary Information. It should be noted that at 500 nm the optical properties are affected by both, the interband transitions of gold and the freely moving electrons. Eq. 1 can still be used if the contribution of the interband transitions can be assumed constant in the range in which the dip moves, which is fulfilled in our case. Figure 4(d) shows how the dip wavelength in the experiment changes with the electrode potential, and Fig. 4(e) shows the change in charge carrier density (relative to that at 0 V) that is estimated from the dip wavelength and Eq. (1). As expected, the charge carrier density does go down for a positive potential and is little affected by a negative potential. For sample 700 the charge carrier density is reduced by 4%, for sample 900 by 8%.

Cyclic voltammetry, such as Fig. 3, records the electric charge which is transferred to the sample. Thereby, the data connects to the change in charge carrier density. Yet, a precise analysis of such data is prevented by the simultaneous occurrence of charge transfer to ions from solution, either in chemical (Faraday-) reactions or during pseudo-capacitive processes such as the electrosorption of oxygen species. In the interest of an order-of-magnitude estimate of the change in charge carrier density in response to potential changes we work with the well-established value of the capacitance of gold electrodes in aqueous electrolyte, *c* = 40 μF/cm^2^ 37. For the potential interval of our study, 0.9 V, this suggests a change in superficial charge density (per area) of 0.36 C/m^2^ or 0.16 electrons per gold surface atom (assuming a dense packed, (111)-oriented surface). For the sample with the smallest ligament size (sample 900, *L* = 10 nm), this corresponds to a variation in the net, volume averaged electron density by Δ*n/n* = 1.5%, with n = 5.9 ∙ 10^22^ 1/cm^−3^ 38. The variation is somewhat less than what is estimated in Fig. 4(e) based on the displacement of the dip along with Eq. (1), yet the general magnitude agrees. We see this qualitative agreement as support for our analysis.

The reason for the stronger effect on sample 900 is that its ligaments are smaller and hence give rise to a larger surface to volume ratio. Since, as mentioned before, charge carriers are reduced only at the gold surface, at a given potential, a larger surface to volume ratio automatically leads to a stronger change in the average charge carrier density of the whole NPG-structure as compared to a solid gold structure. The tremendous surface to volume ratio in fact is the underlying reason for the exceptional electrochemical sensitivity of the optical properties of NPG. The change of a few percent in charge carrier density we find in our samples is comparable to what has been found in previous studies on other geometries^6^,^26^,^28^. It is remarkable that such a small variation in charge carriers has such a profound effect on the transmission (Fig. 4(f)) where the transmission at 950 nm changes by up to 30% for the sample 900 with the largest surface to volume ratio.

Figure 5 shows a comparison of the change in transmission obtained from the experiment as well as from simulation as a function of the change of the carrier density. The simulated response agrees very well with what is seen in the experiment, validating the usefulness of the wire grid as a NPG model.

The reason for this strong response is the nonlinear dependence of the film transmission on the gold permittivity and with that on the charge carrier density. The strong dependence of the transmission on the charge carrier density is due to the qualitative mechanism described as follows. Due to the negative effective permittivity at longer wavelengths the optical wave is reflected from the NPG surface. However, to satisfy continuity conditions of the electromagnetic fields, there will be an evanescent field in the NPG which, provided the film is thin enough, can partly tunnel through the NPG film. The evanescent wave decays exponentially in the NPG film. The steeper this decay the weaker the transmission and the stronger the reflection. The decay constant is proportional to the square root of effective permittivity and thus dependent on the charge carrier density.

**Figure 1:**
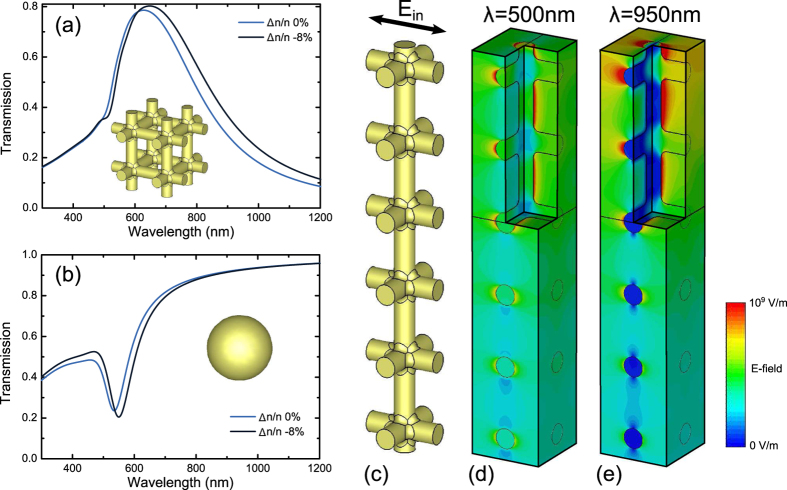
(**a**) Simulated transmission through wire grid with and without changed charge carrier density in gold. (**b**) Simulated transmission through array of gold nanospheres. (**c**) Simulated structure and polarization of the incoming wave for the grid model. The incoming plane propagates vertically from top to bottom and is horizontally polarized as indicated by the black arrow. (**d**) E-field at 500 nm wavelength. (**e**) E-field at 950 nm for the grid model.

**Figure 2:**
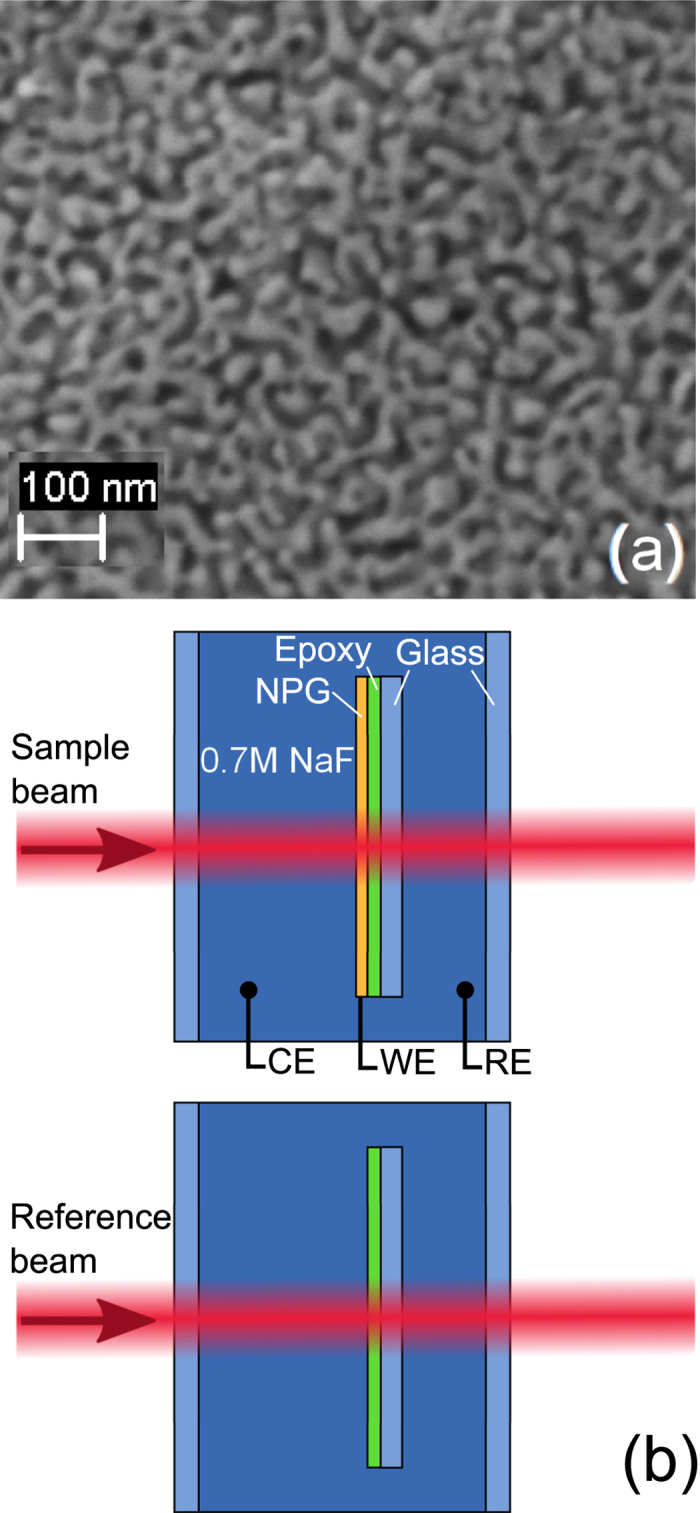
(**a**) Scanning electron micrograph of a typical NPG thin film. (**b**) Experimental setup: Top, the sample is immersed in 0.7 M NaF. The nanoporous gold (NPG) film forms the working electrode (WE). Counter- and reference electrodes are denoted by CE, RE. Bottom, an identical setup but without the NPG film served as optical reference.

**Figure 3:**
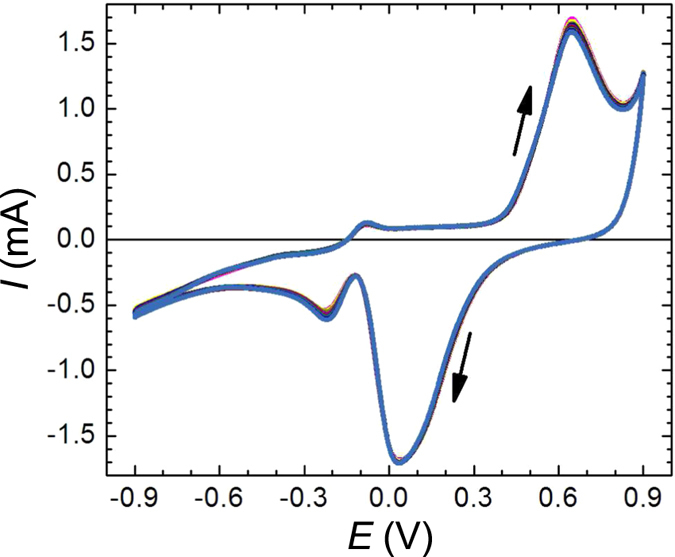
Cyclic voltammetry showing current, *I*, versus potential, *E*, during 10 successive cycles at potential scan rate of 20 mV/s for the example of sample 700. Arrows indicate scan direction. Graphs of the 10 cycles superimpose and cannot be distinguished the scale of the figure.

**Figure 4:**
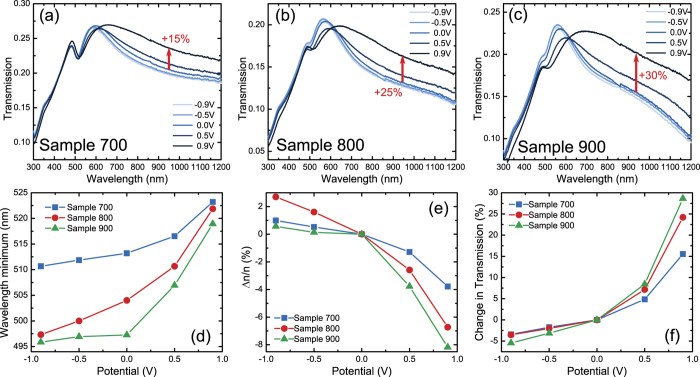
(**a**–**c**) Transmission spectra of NPG with potential cycling from −0.9 V to 0.9 V for samples 700, 800 and 900, respectively. The sample showed weak scattering. Because the spectrometer collected only an unknown fraction of the scattered part of the radiation, the transmission is given in arbitrary units. (**d**) Change in dip position in the data of figure parts a to (**c**). (**e**) Change in charge carrier density relative to the charge carrier density at 0 V, as computed by combining the data from part d with eq. 1. (**f**) Change in transmission at 950 nm. (**d**), (**e**), (**f**) show the averaged measurement data for three consecutive cycles.

**Figure 5:**
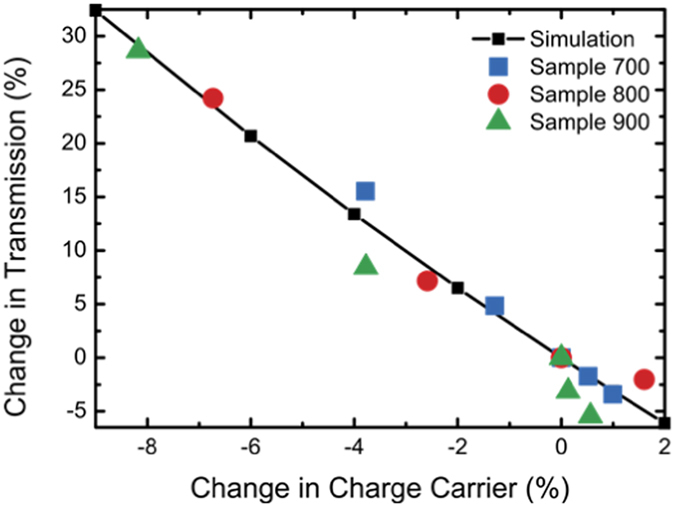
Change in transmission at 950 nm as a function of the charge carrier density obtained from a simulation as well as from experiment.

The NPG film also shows a broadband effect in transmission due to its connected structure. This is different from the effect obtained in gold nanoparticles^25^,^26^,^27^,^28^, where transmission changes are only observed at the resonance frequency (see Fig. 1).

In conclusion, we investigated how the optical properties of NPG change in an electrochemical environment. In our experiment we found that the change in average charge carrier density can be up to 8% if a potential of 0.9 V is applied to the gold. This change in charge carrier density leads to a remarkable 30% near infrared transmission change through an only 200 nm thin layer of material. Based on previous work^18^ we developed a model that can account for the tuning of the dip in transmission at 500 nm as well as the tuning of the transmission for wavelengths longer than 600 nm.

Although the change in charge carrier density is a pure surface effect, the strong response can take place in NPG due to an exceptionally large surface to volume ratio. Our findings agree well with experiments on structures with large surface to volume ratios, however with entirely different topologies, like nanoparticles^26^,^28^ as well as with experiments on surface plasmon polaritons propagating at flat surfaces (SPPs)^6^ where only properties close to the gold surface are probed.

Due to its connected structure and nanoscale porosity NPG in an electrolyte constitutes a novel electro-optic material. We believe that such a strong electrochemical tuning of the optical properties of NPG can have important applications. The exceptionally large effect can be used for the tuning of these optical metamaterials. We consider NPG a novel optical metamaterial platform for the optical sensing of electrochemical processes. We also envisage photoelectrochemical water splitting into hydrogen and oxygen and for solar water purification, where the charge carrier density in the plasmonic structure is an important parameter that controls the efficiency of the process. Compared to other geometries used in that regard, like nanoparticles and SPPs, NPG offers several advantages. Unlike SPPs, NPG doesn’t need a complicated prism coupling scheme and can be evaluated with free space optical transmission experiments. NPG features the same nanometer dimensions with the large surface to volume ratios that make gold nanoparticles interesting as a platform for investigating surface effects. However, contrary to nanoparticles NPG is arranged in a macroscopic film which is electrically connected and thus conductive and allows therefore electrical contact without the help of a conducting substrate. Both NPG and nanoparticles can be investigated via free space transmission experiments, however, per volume, NPG offers a much larger interaction interface compared to a single nanoparticle or a film of nanoparticles. Therefore, the total optical response of NPG will always be much larger for the same change in optical properties of the constitutive metal. NPG also provides the additional advantage of a broadband response in optical transmission, where the nanoparticles have to be characterized at their resonance wavelength.

## Additional Information

**How to cite this article**: Jalas, D. *et al*. Electrochemical tuning of the optical properties of nanoporous gold. *Sci. Rep.* **7**, 44139; doi: 10.1038/srep44139 (2017).

**Publisher's note:** Springer Nature remains neutral with regard to jurisdictional claims in published maps and institutional affiliations.

## Supplementary Material

Supplementary Information
