# Supplementary material for: Electrochemical tuning of the optical properties of nanoporous gold
Source: Sci Rep. 2017 Mar 9;7:44139. doi: 10.1038/srep44139 (PMC5343422; doi:10.1038/srep44139)
Supplement: Supplementary Information [file srep44139-s1.doc]

Electrochemical tuning of the optical properties of nanoporous gold

D. Jalas, L.-H. Shao, R. Canchi, T. Okuma, S. Lang, A. Petrov, J. Weissmüller, and M. Eich

Supplementary information

**Optical simulations**

The simulations in Figure 1 and 5 were done with the frequency domain solver in CST Microwave Studio 2016. For the optical properties of the gold the Drude-critical points model from Ref. 1 was used which takes the free electron effects as well as the interband absorption into account. For the bulk properties the parameters from that reference were used. For properties after change in charge carrier density the paramaters remained the same except for the plasma frequency which was scaled according to , where $n$is the bulk charge carrier density and $\tilde{n}$ is the altered one. The surrounding dielectric had a refractive index of 1.33. Open boundaries were used at the top and the bottom of the structure. On the right and left boundary planes the tangential E-field was set to zero and the tangential H-field was set to zero on the front and rear boundary planes. These mirror boundary conditions resemble a periodic infinite film of the structure.

Just like in the experiment, our model contained 25 mass% gold. The gold wires were chosen to have a diameter of 10 nm and the lattice period was 35.75 nm. To prevent artifacts, we rounded the edges where two wires meet to a radius of 3 nm. The structure was 200 nm high.

The sphere in Figure 1(b) had a diameter of 60 nm and its lattice period was 90 nm.

**Derivation of equation (1)**

In reference 2 we developed an effective medium model for the effective medium dielectric properties of NPG. The model was developed assuming a cubic gold wire grid, where the wires which are parallel to the optical polarization give a different optical response compared to the wires orthogonal to it. The resulting effective permittivity is

$\varepsilon_{eff}=\frac{f_{Au,\perp}\varepsilon_{Au}\frac{2\varepsilon_{d}}{\varepsilon_{Au}+\varepsilon_{d}}+f_{Au,||}\varepsilon_{Au}+f_{d}\varepsilon_{d}}{f_{Au,\perp}\frac{2\varepsilon_{d}}{\varepsilon_{Au}+\varepsilon_{d}}+f_{Au,||}+f_{d}}$. (S1)

Here, $\varepsilon_{Au}=\varepsilon_{Au}^{/}+i\varepsilon_{Au}^{//}$and $\varepsilon_{d}$are permittivities of the gold and the surrounding dielectric. The volume fractions of the parallel and orthogonal gold wires as well as the volume fraction of the dielectric are denoted as$f_{Au,\perp}$, $f_{Au,||}$ and $f_{d}$.The dip in transmission is located at the local maximum of absorption which is the local maximum of the imaginary part of $\varepsilon_{eff}$. As a first step we want to find the maximum of $\varepsilon_{eff}^{//}$ with respect to $\varepsilon_{Au}$. We do this by deriving $\varepsilon_{eff}^{//}$ with respect to the real part of $\varepsilon_{Au}$and equating the derivative to zero. The derivative with respect to $\varepsilon_{Au}^{/}$ yields the following condition:

$\varepsilon_{Au}^{/}\left(\omega_{dip}\right)=-\left(1+2\frac{f_{Au,\perp}}{f_{d}+f_{Au,||}}\right)\varepsilon_{d}$. (S2)

Only the left hand side of equation (S2) is dependent on the charge carrier density. Further, we assume that $\varepsilon_{d}$is only very weakly dispersive and therefore the right side can be assumed frequency independent. To estimate how the position of the dip is altered with the charge carrier density we model the real part of the gold permittivity with the Drude model and one additional term accounting for the interband transitions of gold^3^

$\varepsilon_{Au}^{/}\left(\omega\right)=\varepsilon_{\infty}-\frac{\omega_{p}^{2}}{\omega^{2}+\gamma^{2}}+\varepsilon_{IB}^{/}\left(\omega\right)$, (S3)

with a high-frequency limit dielectric constant $\varepsilon_{\infty}$, the plasma frequency of gold $\omega_{p}$, the collision frequency $\gamma$ and $\varepsilon_{IB}^{/}\left(\omega\right)$ accounting for the interband transistions. The squared plasma frequency is proportional to the charge carrier density $n$ with $\omega_{p}^{2}=\frac{ne^{2}}{\varepsilon_{0}m}$, where $e$and $m$ are the electron charge and mass and $\varepsilon_{0}$is the vacuum permittivity. In the region of the transmission dip around 500 nm the real part of $\varepsilon_{IB}\left(\omega\right)$ changes less than one percent^3^ and can be assumed constant.

To find how the position of the dip ($\omega_{dip}$) changes with the plasma frequency we insert equation (S3) into (S2).

$-\frac{\omega_{p}^{2}}{\omega_{dip}^{2}+\gamma^{2}}=-\left(1+2\frac{f_{Au,\perp}}{f_{d}+f_{Au,||}}\right)\varepsilon_{d}-\varepsilon_{\infty}-\varepsilon_{IB}^{/}$. (S4)

The right hand side of equation (S4) can be assumed independent of wavelength. Accordingly, we can conclude that $\omega_{p}^{2}$is proportional to $\omega_{dip}^{2}+\gamma^{2}$. Further, for 500 nm $\omega^{2}>>\gamma^{2}$ holds,^3^ such that

$\omega_{p}^{2}\sim\omega_{dip}^{2}\sim\frac{1}{\lambda_{dip}^{2}}\sim n$. (S5)

This was used to calculate the charge carrier density change with equation (1).

**References**

1. Vial, A. & Laroche, T. Comparison of gold and silver dispersion laws suitable for FDTD simulations, *Appl. Phys. B* **93,** 139-143 (2008).

2. Jalas, D. *et al.* Effective medium model for the spectral properties of nanoporous gold in the visible, *Appl. Phys. Lett.* **105,** 241906 (2014).

3. Etchegoin, P. G. Le Ru, E. C. & Meyer, M. An analytic model for the optical properties of gold, *J. Chem. Phys* **125,** 164705 (2006).
